# The Dynamics of Cross-Sector Collaboration in Centralized Disaster Governance: A Network Study of Interorganizational Collaborations during the MERS Epidemic in South Korea

**DOI:** 10.3390/ijerph19010018

**Published:** 2021-12-21

**Authors:** Minyoung Ku, Ahreum Han, Keon-Hyung Lee

**Affiliations:** 1 Department of Public Management, John Jay College of Criminal Justice, The City University of New York, New York, NY 10019, USA; 2 Department of Health Care Administration, Trinity University, San Antonio, TX 78212, USA; ahan1@trinity.edu; 3 Askew School of Public Administration and Policy, Florida State University, Tallahassee, FL 32306, USA; klee2@fsu.edu

**Keywords:** disaster governance, public health, network management, collaboration, social network analysis

## Abstract

The debate continues as to which governance structure is most appropriate for collaborative disaster response, particularly between centralization and decentralization. This article aims to contribute to this debate by analyzing the structural characteristics of a multisectoral network that emerged and evolved under strong state control during the 2015 outbreak of Middle East respiratory syndrome-coronavirus (MERS) in South Korea. This study particularly focuses on the evolution of intra- and inter-sectoral collaboration ties in the network. The data for the study were collected through a content analysis of government documents and news articles. Using social network analysis, the authors found that the network evolved into a centralized structure around a small number of governmental organizations at the central level, organizing the ties between participating organizations rather hierarchically. The network displayed a preponderance of internal ties both among health and non-health organizations and among public and nonpublic health organizations, but under different influences of structural characteristics. This tendency was intensified during the peak period. Based on these findings, the authors conclude that the centralization of disaster management may not or only marginally be conducive to cross-sector collaboration during public health disasters, calling for a careful design of governance structures for disaster response.

## 1. Introduction

The COVID-19 pandemic and the history of infectious diseases have reinforced the importance of cross-sector collaboration in containing and responding to public health disasters. Cross-sector collaboration refers to “the linking or sharing of information, resources, activities, and capabilities by organizations in two or more sectors to achieve jointly an outcome that could not be achieved by organizations in one sector separately” [1] (p. 44). In times of health-related disaster, particularly those caused by infectious disease outbreaks, the demand for emergency health and medical services, such as patient diagnosis, transfer, and treatment, as well as the distribution of supplies and equipment, waste treatment and disposal, transportation, childcare, and remote learning could surge, revealing the vulnerability of communities. Collaborations between public and nonpublic sectors and between health and non-health sectors are therefore imperative to protect public health during disasters. These collaborations also increase community resilience and sustainability by gaining control over situations that produce uncertainty and complexity [2]. In countries that rely heavily on the private sector to meet health goals, such as Germany, Japan, South Korea, the Netherlands, and the US, regardless of their regime type, public health’s collaboration with medical care providers and health care facilities is especially vital to address health risks, which prevents health systems from becoming overwhelmed [3]. Multiple studies have also called for breaking down the silos between the fields of public health and emergency management by stressing that public health protection is key to minimizing the impacts of all types of hazards and risks [4,5,6]. 

A well-integrated governance structure can make it easier to solve complex problems that arise in disaster situations, by tapping into networks of organizations that provide necessary services in a collaborative manner. Such a structure as a collection of diverse interdependent organizations (and individuals) can enable early intervention to prevent or reduce the risk of disease spread by pooling and allocating resources [2]. In addition, it helps coordinate action towards shared goals, facilitates learning through interaction, and allows spontaneous exchanges of information, personnel, and services in quick response to changing demands [1,2,7,8,9]. However, cross-boundary collaboration is a challenging process. Disaster management researchers have identified a number of obstacles to collaboration across organizational and sectoral boundaries in times of disaster, including actors’ demotivation and cognitive limitations, lack of proper leadership and management skills, and power struggles within and between professional fields [4,5,6,7,8,9,10]. Those who take an institutional approach have argued that layered upon these challenges are the institutional constraints that shape the actors’ collaborative behavior [4,6,8,9,10]. 

Within disaster management theory, there has been a debate about the appropriate governance of disaster response activities. This debate centers on the question of whether centralization or decentralization is most appropriate for coordination and collaboration problems in turbulent environments, along the line of research on public governance and management. Centralization (or decentralization) generally refers to the extent to which decision-making authority or power is concentrated on a single point. Some claim that centralized systems are effective in managing a surge in demand for a range of services including health care and public safety services in disaster situations to restore and maintain order and stability [11,12,13]. They hold that the government’s central control can ensure clear and timely communication and efficient management of information and resources under turbulent conditions, by prioritizing tasks and eliminating redundant transactions. Others, on the other hand, argue that centralized systems which function on the basis of bureaucratic command and control are rigid and unresponsive or slow to respond to changing needs [7,8,9,14,15]. This perspective advocates for the decentralization of disaster management and its virtues in forming flexible and adaptable response networks in which emergent and multilateral communications and interactions take place among diverse actors. It is based on the belief that organizations can generate innovative solutions to unprecedented challenges through mutual cooperation, by organizing or re-organizing their activities under volatile conditions. 

Despite the ongoing shift towards decentralization, centralized structures have still served as the primary framework for disaster management in many countries. In the context of disaster response, centralization can be conceptualized in two forms: functional centralization and intergovernmental centralization. In a functionally centralized structure, response efforts of organizations that operate in a relatively parallel manner are aligned with the commands of a dominating authority. For example, in the incident command system (ICS), which is widely used in North America and in some other parts of the world (e.g., Australia, Belgium, New Zealand, and the UK), an incident commander or unified command team in the public sector is responsible for directing and coordinating response operations [11,12,13]. Although the system is founded on the principles of bureaucracy (i.e., standardization, formalization, specialization, and hierarchy), under the notion that all disasters are local, a local authority (e.g., a local government fire chief arriving on the incident site first) is to fill the commander’s position [11]. The role that federal or central government plays in the system is limited to supporting state or local agencies, though in some countries such as the UK there is still room left for central government control over response to multi-site incidents [11,16]. In a vertically centralized system, however, control over disaster planning and response is held by the central government, while operational functions are performed to varying degrees at the local level. In China and Turkey, for example, central authorities tend to govern strategic, tactical, and occasionally even operational decisions during disasters [17,18]. In the Netherlands, Sweden, and Denmark, network approaches are integrated into disaster management, yet making strategic decisions to coordinate responses to large-scale disasters remains in the hands of central government [16]. 

The capability and effectiveness of centralization, whether functionally or vertically centralized, in managing public health disasters has yet to be conclusively demonstrated. Some have argued, based on evidence from the use of ICS at local incidents, that a centralized system can produce a scalable and flexible structure of collaboration in response to a disaster [11,13]. Recently, a similar claim has been made for the effectiveness of state intervention to facilitate public-private partnerships in response to public health disasters such as COVID-19 [14]. Yet, others have questioned the adequacy of centralized government control to promote cross-sector collaboration under complex, uncertain, and time-pressured situations. Specifically, researchers have raised concerns about the assumptions underlying a centralized system of disaster response, including pre-specified roles and responsibilities of participants; particularized training provided before implementation; shared understanding of the system among the participants; and a standardized course of action to deal with every disaster situation [9,15,16]. With reference to interorganizational collaboration, they point to difficulties when integrating unplanned actors (particularly non-governmental actors) and relationships into the system as new situations emerge; gaps in familiarity and knowledge of the system between emergency mission organizations (e.g., police and fire, and emergency management) and other organizations (e.g., hospitals, social services, and public works); and a bottleneck of communication at the peak of incident intensity [6,7,8,9,16,19,20]. They are especially skeptical of the capacity of organizations in the system to share timely and accurate information and feedback which is essential to flexible and adaptive coordination [21]. These are seen as structural flaws inherent in centralized systems, rather than the consequences of managerial incompetence [19]. 

However, these claims have been subject to the criticism that they lack sufficient evidence [22]. Moreover, most of the handful of empirical works on health-related disaster management have focused on the design and operation of the ICS, which is more functionally centralized, or on the position and role of health professionals in the system [12,13]. Political and administrative centralization amid public health disasters has become a critical issue in disaster governance, yet little attention has been paid to its effect on interorganizational collaboration, particularly on the dynamics of cross-sector collaboration under strong state control. 

In the present study, therefore, we examine the influence of state-led centralization on cross-sector collaboration, among government, civil society, and private sectors, in response to a public health disaster from an evolutionary perspective, within the context of South Korea (hereafter, “Korea”)’s response to the 2015 MERS outbreak, by answering the following questions: (1) How was the network of organizations that responded to the epidemic shaped over time?; (2) How did collaborations between health and non-health sectors and between public and nonpublic health sectors unfold in the network? In particular, how did the ratio of intra- and inter-sectoral collaboration ties change during the response phases?; and (3) How did the structural characteristics of the network influence these patterns of cross-sector collaboration?

Our aim is to contribute to the literatures on disaster management and public governance by adding empirical evidence to the debate on whether centralized or decentralized governance structure is more effective in facilitating cross-sector collaboration to solve complex problems that arise during public health disasters. In this paper, we particularly focus on the structural dynamics of response networks that emerge under strong state control and on the evolutionary patterns of interorganizational collaboration in the networks within and across sectors in response to disasters caused by infectious diseases. To do so, we employ social network analysis to analyze the data on interorganizational responses to the MERS epidemic in Korea. Social network analysis allows researchers not only to capture and visualize the structure of social networks, but also to analyze and understand the patterns of interactions and relationships between actors underlying the structure. We, therefore, empirically show that a nationwide response network can evolve dynamically during a large-scale public health disaster, such as an epidemic or a pandemic, in a country that adopts a centralized national disaster management system, using social network data and methods. Our findings suggest, however, that strong government control over response activities may constrain the innovative capability to deal with unprecedented threats and challenges through dynamic collaborations among organizations across sectors in the network.

MERS, first having emerged in the Arabian Peninsula in 2012, entered Korea through a traveler in May 2015 and rapidly morphed into an epidemic in early June of the same year. Korea has a two-tier system to manage health-related disasters. As of 2015, Korea’s Framework Act on the Management of Disasters and Safety (FAMDS), which defines the country’s emergency management system, granted general authority to control and coordinate disaster management affairs at the national and subnational levels to the Ministry of Public Safety and Security (MPSS) (Chapter I, Article 6). The Ministry of Health and Welfare (MOHW) had the authority and responsibility to prevent and control infectious diseases under the Infectious Disease Control and Prevention Act (IDCPA). During the epidemic, the MOHW thus took the lead in organizing health-related measures (e.g., epidemiological investigation, patient treatment, laboratory testing, and health care resource allocation), and the MPSS coordinated non-health-related responses, under the supervision of the Prime Minister. Despite the national emergency plans for infectious diseases, including MERS, considerable coordination challenges arose throughout the response process as the number and diversity of participants and activities sharply increased [23]. This outbreak, as the second largest MERS epidemic in the world recorded to date, resulted in 186 cases and 38 deaths by late December 2015 [24].

## 2. Materials and Methods

We used social network analysis approaches to examine the emergence and evolution of Korea’s MERS response network, focusing on the patterns of interorganizational collaboration across sectors, from a network structural perspective. A network can be defined as a collection of three or more nodes (e.g., individuals, teams, and/or organizations) that work together to achieve a collective goal [10]. To collect data on the network of organizations that participated in response to the 2015 outbreak, we classified network ties into four categories that define collaborative relationships, given the theoretical discussion in the previous section: communication, information sharing, resource sharing, and joint action or decision making. Each of the relational categories is defined as follows: (1) communication as sending a verbal or written message to convey a request, command, complaint, or suggestion; (2) information sharing as transmitting any data or message based on fact or professional expertise relevant to the epidemic; (3) resource sharing as providing money, services, personnel, or physical materials; and (4) joint action or decision making as the action or decision that two or more nodes (i.e., organizations) take or make together. These categories, particularly communication and information sharing, are not necessarily mutually exclusive. The ties were then separated into three time periods corresponding to the Korean government’s response phases [23]: early response (from 20 May to 8 June ), active response (from 9 June to 27 July ), and post-response (from 28 July to 23 December ). The response phases are characterized in terms of epidemic progress, respectively, by the introduction and localized transmission of the virus; by amplification and reduced transmission of the virus; and by resolution of the crisis situation [2]. 

To identify the organizations that participated in each of the response phases and the ties that connected them in each of the relational categories, we conducted a content analysis of government documents and news articles. The data sources were a white paper published by the MOHW in 2016, and 6187 news articles published by four major domestic newspapers (i.e., Kyunghyang Shinmun, Hankyoreh Shinmun, Dong-a Ilbo, and Hankook Ilbo) between 20 May and 31 December 2015. The white paper contains 1125 pages that describe intra- and interorganizational activities that occurred during the epidemic, including copies of press releases and interoffice memoranda issued by government authorities. The selected newspapers reflect a diverse political spectrum from right to left. In doing so, we reduced the chances of any groups of organizations being overrepresented in the data by their political stance. Three investigators, including the first author of this paper, collected, coded, and cross-checked the data by manually reading them. First, they independently collected information about “who did what, with whom, when, and how” from the sources. To address the discrepancies between the independently coded data sets such as different organization names, different descriptions of relational events, and missing data entries, two of the three investigators re-coded the problematic sources and double-checked data coding accuracy independently first and collectively later to increase the reliability of the data. Depending on the relational type, data records were labeled as “communication”, “information sharing”, “resource sharing”, or “joint action or decision making”. In this process, we eliminated the data that fell into none of the four categories or did not contain time information. The processed data were converted into sociomatrices using UCINET 6.720 (Analytic Technologies, Lexington, KY, USA) [25]. In the sociomatrices, nodes represent organizations and ties indicate uni- or bi-directional communication, information sharing, or resource sharing; or bilateral action or decision making in the early response, active response, or post-response phase. Thus, ties in the “joint action or decision” matrix are symmetric, while ties in the other matrices are symmetric or asymmetric. The sociomatrices have only binary values, indicating the presence (“1”) or absence (“0”) of ties from one node to another node. We also collected information about the sectoral affiliation of the nodes from the organizations’ websites. 

We employed social network methods to analyze and visualize the data, using UCINET 6.720 [25]. To capture the evolutionary characteristics of the network structure, some descriptive statistics were calculated for the whole network in each phase. Among them, in- and out-centralization measures assess the extent to which ties are focused around one or few nodes in the network, taking into account the direction of the ties. To identify influential nodes, we measured out-degree centrality, which counts the number of ties directed out of a node. The centrality scores reported in the study were normalized, controlling for network size effects as the numbers of nodes differed across the response phases. Mathematical formulas for the measures can be found in Wasserman and Faust [26].

Krackhardt’s graph theoretical dimensions of hierarchy (GTD) analysis was carried out to capture the degree of structural hierarchy in the network, that is, the degree of similarity to an out-tree in which any two nodes are connected by one tie pointing towards the root. Krackhardt proposed four GTD measures which assess the extent to which a network structure deviates from pure hierarchy [27]: connectedness, graph hierarchy, graph efficiency, and least upper boundedness (LUB). These measures take a value between 0 and 1. In a pure hierarchy, every node reaches another without an isolated node or an isolated group of nodes (i.e., connectedness = 1). When a directed network in which arrowheads indicate the direction of command is purely hierarchical, ties in the network are not reciprocated (i.e., graph hierarchy = 1). A purely hierarchical network has the highest efficiency of 100 percent because there are no redundant ties (i.e., graph efficiency =1). In a network of N nodes, the network efficiency thus decreases as the number of dyads increases greater than N-1. All pairs of nodes in the pure hierarchy, except the one at the top, have a common node from which paths that terminate to them emanate (i.e., LUB = 1). Mathematical formulas for the measures can be found in Krackhardt [27].

The E-I index model proposed by Krackhardt and Stern, used to determine whether, and the extent to which, collaborations occurred across sectors in Korea’s MERS response network [28], is as follows:

$$E\text{-}I\;\mathrm{index}=\frac{EL-IL}{EL+IL}$$

where *EL* = the number of external links between nodes in different groups, and *IL* = the number of internal links between nodes in the same group. The scale varies from +1 (i.e., completely external ties) to −1 (i.e., completely internal ties) based on a specific attribute. In this study, nodes were partitioned according to their affiliation with the health and public sectors. It should be noted that the comparisons by public and nonpublic (i.e., for-profit, and nonprofit) nodes were performed only on health nodes as collaboration between public health agencies and health care providers and research institutions outside the public sector during the epidemic is of interest of this study. 

The E-I model incorporated in UCINET 6 produces, in addition to the observed value, a range of possible values of the E-I index, based on the number of groups, group size, and the total number of ties in the network (i.e., the network density). This allowed us to examine in two ways whether state-led centralization of response efforts facilitated or hindered cross-sector collaboration during the MERS outbreak, from a network structural perspective. First, using an expected E-I index, we investigated whether the tendency for within- or cross-sector collaboration was expected on average to appear by chance alone in the network, given the sector size and the network density, when the nodes were dichotomized by sector. In the previous section, we considered that the centralization of governance structures may affect, if not determine, who enters a response network and how flexible it can be to create ties between them. That is, by controlling or influencing the basic conditions of network formation, the government’s policy regarding centralization or decentralization of response may intentionally or unintentionally create an environment in favor of or against cross-sector collaboration in times of disaster. An expected value of E-I index thus measures the degree of “network structural effects”. Second, nodes in the network may show a higher focus on internal or external ties than the random distribution. To test for the existence of “preference effects” that go beyond the network structural effects, we assessed whether an observed E-I value was significantly different from the expected one by a permutation test. Since the E-I index models did not consider the direction of ties, symmetrized networks were used in the two sets of analyses. 

## 3. Results

Table 1 summarizes the descriptive statistics of Korea’s MERS response network. The introduction of the novel virus prompted the participation of more than 1200 nodes in response operations. A majority of them were non-health nodes, and all of the non-health nodes, except six, were public sector organizations, especially those providing police, fire, and emergency services. The proportion of health nodes in the population increased between phases and reached 84% in the post-response phase. The vast majority of the health nodes in the early (79%) and active (83%) response phases were also in the public sector, but this was reverted when privately owned hospitals and clinics emerged to become major players in the post-response phase, accounting for 55% of the nodes. More than half of these nonpublic health care providers were new actors that joined local health networks created by the government for local capacity building after the epidemic peak. Although nodes were allowed to both join and leave the network between phases, the network size decreased, with the substantial withdrawal of non-health organizations across the response phases and public health organizations between the active response and post-response phases. 

The network became denser over the phases, but the network density was still less than 1%, indicating a low level of collaboration across organizations. Communication was the primary reason that the nodes were connected to other nodes in all the three phases, followed by information sharing in the early response phase. Only a small proportion of nodes in the network were engaged in resource sharing in the early phase, but resources came to play more of a part in shaping collaboration ties in the network as the epidemic proceeded. The nodes were most active in making decisions or taking joint action in the early phase, whereas it decreased during the peak as well as the fall of the epidemic. 

A small world phenomenon was also observed in the network. The average geodesic distances indicate that the nodes were connected to one another on average through two to four other nodes. The network average clustering coefficient, which measures the degree to which nodes in a network cluster together, was low (0.25) in the early phase, but was more than doubled in the active response (0.53) and post-response (0.58) phases. These results suggest that although the network shrank in size and showed limited compositional variation between the phases, the network evolved, though slowly, stimulating network cohesion among nodes. 

Graph centralization and four GTD scores in Table 1 indicate that the network was characterized by persistent hierarchy and growing centralization. The out-centralization score was only 0.26 in the early response phase but rose to 0.57 in the active response phase and again to 0.85 in the post-response phase. The GTD scores were invariably high in all the phases (>0.80), suggesting that the centralization of response efforts in the network might have been predicated on hierarchical relations between participating organizations. 

**Figure 1:**
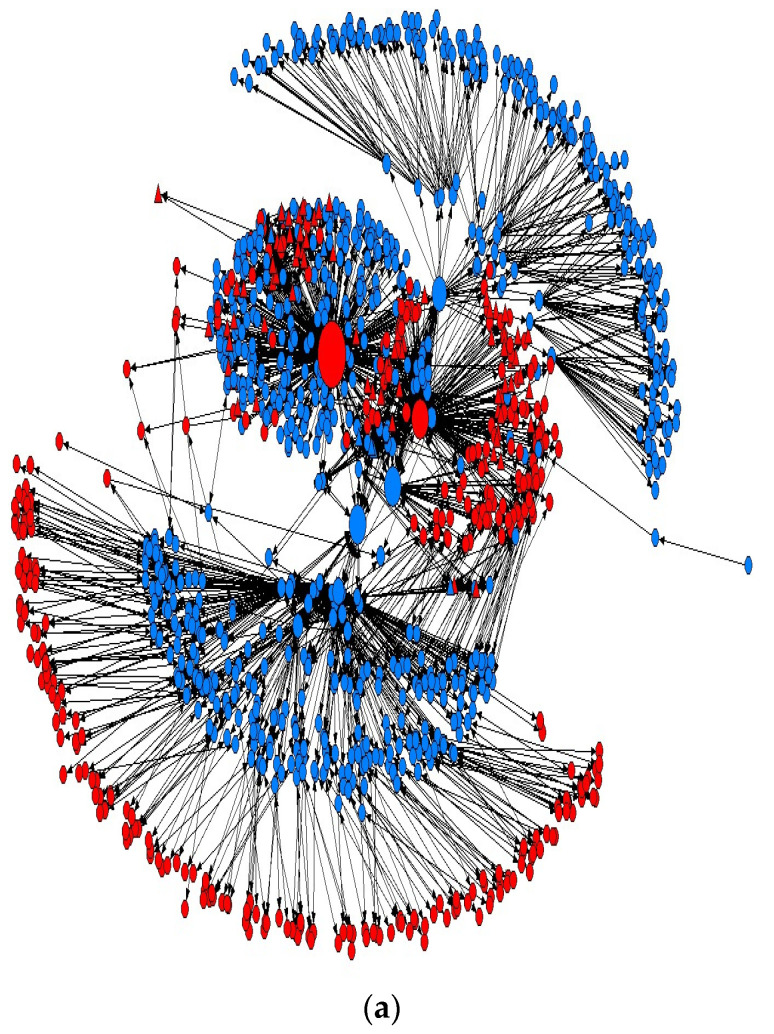

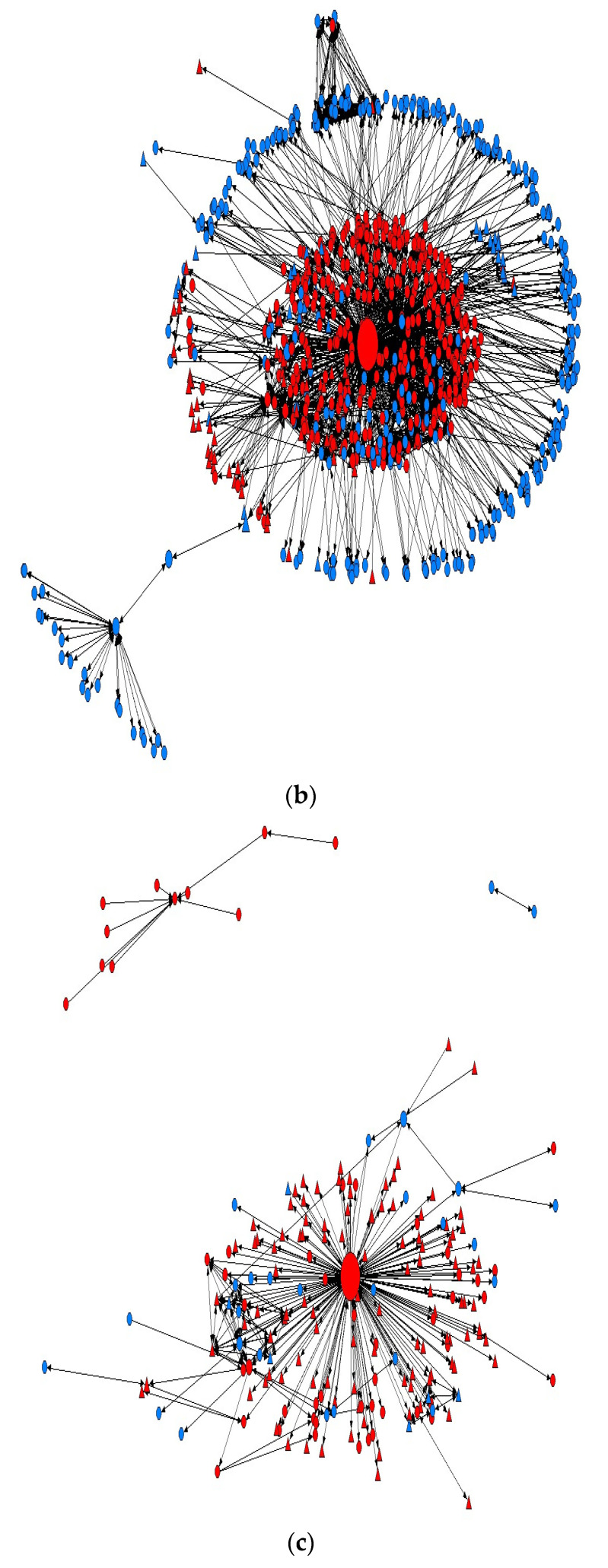
The evolution of Korea’s MERS Response Network. The visualization of network structures (**a**) in the early response phase; (**b**) in the active response phase; and (**c**) the post-response phase. Red nodes represent health organizations, blue nodes nonhealth organizations. Circles are public organizations, up triangles nonpublic organizations. The size of the nodes reflects their betweenness centrality, which counts the number of shortest paths that pass through the node to link between any other nodes.

Table 2 demonstrates that the central government took the lead in coordinating response activities in the network. All of the most influential nodes in the out-communication networks, except two, were in the public sector at the central level. The dominance of central government, particularly of the MOHW, peaked during the active response period as its normalized out-degree centrality was nearly doubled from 0.24 to 0.47. The increasing out-centralization of network structure and the strong state steering, illustrated by the out-communication centrality scores of the nodes, together confirm that the vertical centralization of response efforts was in play during the epidemic. The network evolution is visualized in Figure 1.

Table 3 reports the group-level E-I index test results regarding the internal/external ratio of collaborative ties in the network. The expected values for all layers of the whole network and the health only network in all three phases were negative, indicating that the structural conditions of Korea’s MERS response network were more favorable for internal collaborations. Given the sector sizes and the network density, the nodes were thus predicted to collaborate with other nodes more from the same sector, when dichotomized by health and non-health sectors and by public and nonpublic sectors. This structural effect was particularly expected to be strong in collaboration between public and nonpublic health nodes in the early and active response phases and in collaboration between health and non-health nodes in the post-response phase. 

The results of the permutation tests in Table 3 confirm the overall preponderance of internal ties in the network especially during the response period, but a mix of structural and preference effects on the patterns of collaboration was observed. In the early response phase, the observed E-I index values for the ties in the whole network (i.e., all nodes were considered and partitioned between health and non-health sectors) and the health only network (i.e., only health nodes were considered and partitioned between public and nonpublic sectors), except the “joint action or decision” ties in the whole network, were negative. However, statistically significant differences between the observed and expected values were observed only in the whole network (*p* < 0.05). A similar pattern was identified in the active response phase, as the observed values for the ties in the whole network and the health only network, except the “resource sharing” ties in the whole network, were negative. In this phase, the models for all types of ties in the whole network and for the “communication” and “information sharing” ties in the health only network detected significant differences between the observed and expected values.

The results show greater variation in the post-response phase. Overall, the prevalence of internal ties among health and non-health nodes remained intact, whereas ties among public and nonpublic health nodes moved into focus on external ties. The observed E-I index value for the whole network, as an aggregated structure of all types of ties, was negative, though the observed values for some subsets of ties (i.e., communication and information sharing) in the network were slightly positive. However, the observed values, except the value for the “resource sharing” ties in the whole network, were not significantly different from the expected ones. Unlike the whole network, the observed values for the ties in the health only network were consistently positive, and the positive value for the aggregated network structure comprising all the four types of ties among the health nodes was significantly different from the expected one. These, combined with the results reported above, suggest that under strong state control, collaboration rarely occurred across organizational lines in the response and post-response periods and that when it did, it tended to occur more within the sectors. This tendency observed in the health only network might have been related to structural properties of the network, particularly to the difference in size between public and nonpublic sectors, while other factors might have played more into the collaborations between the health and non-health nodes. 

## 4. Discussion

This study has investigated how the structure of a network representing collaborative relationships between organizations to communicate, share information and resources, and coordinate joint actions and decisions can emerge and evolve under centralized control in times of public health disaster. Using social network analysis to analyze the data on interorganizational responses to the MERS epidemic in Korea, we have demonstrated that the network may not be completely rigid, but can show more or less responsiveness to changing situations. However, in the Korean case, strong state control over disaster operations led to the centralization of network structure with limited scalability and flexibility. In the network, cross-sector collaborations particularly between public and nonpublic sectors and between health and non-health sectors were limited whereby sectoral clusters grew as the epidemic progressed. Going one step further, our examination of the association of this preponderance of intra-sector collaboration with the structural characteristics of the network has revealed that the centralization of disaster management can facilitate or inhibit collaboration within or across sectors during disasters by shaping a response network in a particular way, e.g., determining the network size and controlling the entrance or exit of nodes in particular sectors. These findings suggest several theoretical and policy implications. 

First, a country’s disaster governance structure influences the structural and compositional characteristics of interorganizational networks that emerge during public health disasters. This effect is likely to lead to a centralized network structure in which decision-making authority to coordinate response activities across organizations and sectors is concentrated in a few actors at the central government. The Korean government adopted the bureaucratic command and control model of disaster management in the 1970s, and efforts have progressed towards an integrated and comprehensive nationwide disaster management system since 2004 [29]. Despite the decentralization reforms in the country around the 2000s, the centralization of state-local relationships in disaster management has persisted, and the position of civil societies and private sectors in disaster governance has been weak [29]. This was manifested in the centralized structure of the collaboration network that emerged during the MERS epidemic. The network was characterized by loose connections between nodes, high levels of hierarchy, group clustering, and short paths. These structural properties remained relatively constant while network centralization increased towards the peak of the epidemic. 

The network displayed greater variation in composition than in structure across the response phases in the Korean case, as indicated by the differences in the numbers and proportions of sectoral nodes. The network size, however, consistently decreased with increased centralization of control over the response operations. Notwithstanding that the proportion of public hospital beds was only around 10% as of 2015 so that the engagement of healthcare providers and partners in the private sector was essential for successful response to the epidemic [3], the participation gap between public and nonpublic health organizations was reinforced through the response process. These findings, with the fact that the health authorities modified the response manual multiple times to resolve coordination problems in the early and active phases of response [23], suggest that as previous research on disaster and emergency management networks has claimed [19,30], a response network that emerges under centralized control may not be flexible enough to incorporate a wide range of new actors and relationships into the system beyond the scope of the plan. Moreover, when inclusion occurs, the relationships between participants may be more hierarchically rather than functionally organized.

Second, the results of our E-I index analysis support the contention that strong state control over response efforts may limit the capacity of organizations to tackle complex problems through cross-sector collaborations during public health disasters. The results further show that vertical centralization can even exacerbate existing communication, information, and resource disparities between sectors. A hierarchical and centralized model of disaster management may not necessarily hinder interorganizational collaboration under volatile conditions but rather the opposite may be expected, as suggested by the increasing clustering coefficients on the MERS response network over the phases. This effect was, however, seen mostly among in-group participants, and participants were constrained and limited in their capacity to collaborate across health and non-health sectors during the response operations. The same tendency was observed when health participants collaborated across public and nonpublic sectors, and this pattern was accentuated for communication and information sharing during the peak period. 

Seamless and timely communication among diverse actors has been noted to be of great importance during an epidemic response to reduce the risk of spreading infections in the early stage of the epidemic as well as manage the surge of patients in the active stage through spontaneous collaboration [2,21]. However, many disaster management researchers have acknowledged the limited capacity of organizations in centralized systems to communicate and share information and feedback which is key to the process of mutual adaptation and coordination [7,8,9,16,19,20,21]. Our findings confirm this concern. In the Korean case, the limited sharing of communication and information between public health authorities and private entities, particularly the hospitals where early MERS patients sought medical treatments, led to a significant delay in the diagnosis and isolation of the first patient, causing transmission in and between hospitals. These difficulties evolved into communication and information sharing problems across health and non-health agencies in the public sector, resulting in a lack of knowledge or misunderstanding about the virus. Consequently, some local governments refused to accept MERS patients to the hospitals in their jurisdiction or ignored patient transfer requests from outside their jurisdiction, heightening both state-local and local-local tensions [31,32]. 

Although some scholars have argued for the scalability and flexibility of interorganizational response to disasters under a centralized system, many others have pointed out that this may occur at best under limited conditions. The positive and significant values of the E-I index for joint action or decision in the early response phase and for resource sharing in the active response phase, in terms of collaboration between health and non-health sectors, may be interpreted in line with this argument. The Korean government has mandated the creation of the Infectious Disease Control Committee, which is cross-sectoral, at the national level since 2010 to better manage infectious diseases through collaborative governance (the IDCPA, Chapter II, Article 9). In addition, Korean public health law stipulates the reasonability of medical institutions and personnel to cooperate with governments at all levels in surveillance and control of infectious diseases (the IDCPA, Chapter I, Article 5). Similarly, the country’s disaster management law also mandates in general terms for governmental and nongovernmental entities to render assistance upon request to local governments that respond to disasters (the FAMDS, Chapter VI, Section 1, Article 44). We observed from the data that these legal rules operated to convene the cross-sectoral committee and support its activities as well as to mobilize resources from the private sector, especially from private hospitals, during the epidemic. Recent research on Korea’s response to COVID-19 presents the timely provision of supplies, equipment, and services by private firms, local governments, and civil society organizations in the early response phase as evidence that supports the effectiveness of the government’s central control in leveraging resources and expertise through collaborative partnerships [14]. Our results, however, suggest that this may be largely a product of legal compliance rather than of “the spirit of collaboration”. In addition, the results also suggest from a managerial perspective that in a vertically centralized system, governments may be more likely to utilize bureaucratic mechanisms rather than network ones (e.g., shared norms and understandings, trust, and negotiation) to fill the “holes” between sectors, but that collaboration ties enacted through these bureaucratic mechanisms may be only temporarily effective. In other words, adaptive learning which enables organizations to work collaboratively under turbulent conditions may be limited or is unlikely to take place in centralized governance structure under strong state control. 

Third, the challenges of collaboration especially between health and non-health sectors under state control in disaster situations may have deeper roots than accounts of network structure would suggest. A possible account is that professional identities provide organizations and individuals with a frame of reference through which to interpret the world and decide on what course of action to take by shaping their logics and priorities [33]. By helping to develop shared understanding and practice, on the one hand, a shared professional identity can facilitate collaboration among organizations within a domain in times of disaster [34]. The walls between different domains, on the other hand, often raise tensions between participants in disaster response networks. These tensions are particularly acute between emergency mission agencies (i.e., fire and police departments) and public health agencies in bureaucratic structures. Specifically, prior research on public health disaster governance in the US has pointed to the isolation or weak integration of public health perspectives into disaster management systems, calling for integrative approaches at both the policy and practice levels to break the silos and achieve an effective response to increasing health risks [4,5,6]. The results of this study suggest that the findings of previous studies generalize to Korea’s disaster management. 

Overall, our findings are consistent with other studies arguing that a centralized governance structure led by strong government leadership is not conducive to cross-sector collaboration in times of disaster. Moving one step further, we demonstrated that this tendency might cause more difficulties for collaborative problem-solving when an emergency situation is in progress, rather than when it has just happened or been resolved, from an evolutionary perspective. We also showed that these problems might be linked not only to the structural conditions of the network imposed by the centralization of disaster response, but also to deeper social, political, and other mechanisms, and thus legal institutional approaches to collaborative disaster management, such as mandating cross-sector collaboration and cooperation, might have only limited impacts. 

Despite these contributions, our research has some limitations. We dichotomized sectors by coding nodes as “health” if they provided health-related services or programs and “non-health” otherwise. The health nodes were identified as “public health” if they were in the public sector and everyone else identified as “nonpublic health”. This allowed us to reduce analytic complexity, but at the expense of a richer and more nuanced understanding of cross-sectoral collaboration in a centralized system during public health disasters. Future studies may wish to explore the topic with more diverse classifications of sectors. Another limitation is imposed by the nature of our data sources. Although the white paper and news articles enabled us to identify more than 1200 organizations participating in response operations during the epidemic and 2700 ties between them, they tended to focus on formal response activities by government agencies at the central level. This limited our ability to collect information about informal communication and interaction among the organizations as well as formal communication and interaction among local-level agencies and nongovernmental organizations. We suggest that future studies include post-event documents published by local authorities and news articles by local newspapers, though this may not entirely resolve the problem of data comprehensiveness. Since our analysis was conducted on a single case basis, further studies are needed to examine the generalizability to different public health disasters such as COVID-19 and to different countries. 

## 5. Conclusions

Collaboration within and across sectoral boundaries has been identified as essential to the successful management of disasters with greater frequency and intensity. While diverse modalities of disaster governance through the strong state, the entrepreneurial spirit of the public sector, and civil self-government, have been proposed to date, centralized disaster management systems have been widely used in many countries. Their effectiveness in coordinating collaborative responses by streamlining communications, pooling and sharing information and resources, and supporting joint actions and decisions across sectors has, however, been subject to much criticism. This concern is supported by the findings of this study, since vertical centralization strengthened the centralization of network structure around governmental actors who held bureaucratic power. Furthermore, the engagement of nongovernmental actors in the network was limited, and connections between participating organizations was rigid and hierarchical. 

While our study highlights the structural limitations of centralized, bureaucratic systems within the context of public health disaster response, they do not necessarily mean that centralization is inferior to decentralization. Since our analysis focuses only on a centralized disaster management system in a country, more studies are needed to reveal which governance structure, at least between centralized and decentralized structures, is most effective in managing the growing threats to public health. We also believe that answering the question about whether and under what conditions centralized or decentralized disaster governance works best is equally important for understanding the relationship between governance structure and disaster management performance, as well as creating and sustaining resilient societies prepared for future public health disasters. 

## Figures and Tables

**Table 1:** Network Descriptive Statistics.

	Early Response	Active Response	Post-Response
# of Organizations ^1^	1202	785 (87)	194 (74)
• Public health organizations	336	363 (29)	55 (3)
• Nonpublic health organizations	88	73 (16)	107 (61)
• Nonhealth organizations	778	349 (42)	32 (10)
# of Ties ^2^	2780	2459	354
• Communication	1574	987	157
• Information sharing	1465	897	128
• Resource sharing	498	963	143
• Joint action or decision making	1212	676	86
Network Density (%)	0.19	0.40	0.95
Average Geodesic Distance	3.68	3.12	2.27
Clustering Coefficient	0.25	0.53	0.58
Krackhardt GTD Measures			
• Connectedness	1.00	1.00	0.87
• Hierarchy	0.93	0.81	0.90
• Efficiency	0.99	0.99	0.99
• LUB	0.99	0.99	0.99
Network Centralization	0.31	0.59	0.86
• Out-Centralization	0.26	0.57	0.85
• In-Centralization	0.11	0.21	0.15

Note. ^1^ The numbers of nodes that newly entered the network are reported in parentheses. ^2^ Multiple categories of ties were allowed to exist between pairs of nodes.

**Table 2:** Three Organizations of Highest Normalized Out-Degree Centrality in Communication.

Early Response		Active Response		Post-Response	
Organization	Centrality	Organization	Centrality	Organization	Centrality
Ministry of Health and Welfare	0.24	Ministry of Health and Welfare	0.47	Ministry of Health and Welfare	0.17
Korea Centers for Disease Control and Prevention	0.06	National Medical Center	0.08	Korea Centers for Disease Control and Prevention	0.06
Gyeonggi Provincial Police Agency	0.05	Ministry of Environment	0.04	The Korean Society for Preventive Medicine	0.05

**Table 3:** Group-Level E-I Index Analysis for Sector Group.

	Early Response			Active Response			Post-Response		
	E-I			E-I			E-I		
	Observed	Expected		Observed	Expected		Observed	Expected	
Health—Nonhealth									
Whole Network	−0.29 *	−0.09	(0.07)	−0.20 *	−0.01	(0.04)	−0.51	−0.43	(0.33)
• Communication	−0.37 *	−0.09	(0.09)	−0.73 *	−0.01	(0.07)	0.03	−0.43	(0.26)
• Information Sharing	−0.41 *	−0.09	(0.09)	−0.78 *	−0.01	(0.07)	0.11	−0.43	(0.28)
• Resource Sharing	−0.73 *	−0.09	(0.09)	0.35 *	−0.01	(0.05)	−1.00 *	−0.43	(0.45)
• Joint Action or Decision	0.15 *	−0.09	(0.06)	−0.60 *	−0.01	(0.06)	−0.02	−0.43	(0.28)
Public—Nonpublic Health									
Health Only Network	−0.20	−0.34	(0.23)	−0.58	−0.44	(0.35)	0.32 *	−0.10	(0.25)
• Communication	−0.38	−0.34	(0.23)	−0.72 *	−0.44	(0.42)	0.09	−0.10	(0.24)
• Information Sharing	−0.43	−0.34	(0.23)	−0.70 *	−0.44	(0.42)	0.15	−0.10	(0.27)
• Resource Sharing	−0.01	−0.34	(0.31)	−0.25	−0.44	(0.27)	0.36	−0.10	(0.29)
• Joint Action or Decision	−0.51	−0.34	(0.27)	−0.37	−0.44	(0.37)	0.13	−0.10	(0.31)

Note. 10,000 random permutations were performed to compare the observed values of E-I index to those expected by random mixing, given the blocks of groups (i.e., sectors) and the overall tie-densities. For the whole network, both health and non-health organizations are considered, while network data only between health organizations are entered to analyze the health only network. Standard deviations are reported in parentheses. * *p* < 0.05.

## Data Availability

Data sharing is not applicable to this article.

## References

[1] Bryson J.M., Crosby B.C., Stone M.M. (2006). The design and implementation of cross-sector collaborations: Propositions from the literature. Public Adm. Rev..

[2] World Health Organization Managing Epidemics: Key Facts about Major Deadly Diseases 2018. https://apps.who.int/iris/handle/10665/272442.

[3] OECD Stat Health Care Resources: Hospitals. https://stats.oecd.org/index.aspx?queryid=30182.

[4] Colmers J.M., Fox D.M. (2003). The politics of emergency health powers and the isolation of public health. Am. J. Public Health.

[5] Kennedy M., Gonick S., Meischke H., Rios J., Errett N.A. (2019). Building back better: Local health department engagement and integration of health promotion into Hurricane Harvey recovery planning and implementation. Int. J. Environ. Res. Public Health.

[6] Rose D.A., Murthy S., Brooks J., Bryant J. (2017). The evolution of public health emergency management as a field of practice. Am. J. Public Health.

[7] Comfort L.K. (2007). Crisis management in hindsight: Cognition, communication, coordination, and control. Public Adm. Rev..

[8] Drabek T.E., McEntire D.A. (2002). Emergent phenomena and multiorganizational coordination in disasters: Lessons from the research literature. Int. J. Mass Emerg. Disasters.

[9] Neal D.M., Phillips B.D. (1995). Effective emergency management: Reconsidering the bureaucratic approach. Disasters.

[10] McGuire M., Agranoff R. (2011). The limitations of public management networks. Public Adm..

[11] Bigley G.A., Roberts K.H. (2001). The incident command system: High-reliability organizing for complex and volatile task environments. Acad. Manag. Ann..

[12] Burkle F.M., Hsu E.B., Loehr M., Christian M.D., Markenson D., Rubinson L., Archer F.L. (2007). Definition and functions of health unified command and emergency operations centers for large-scale bioevent disasters within the existing ICS. Disaster Med. Public Health Prep..

[13] Quinn E., Johnstone T., Najjar Z., Cains T., Tan G., Huhtinen E., Nilsson S., Burgess S., Dunn M., Gupta L. (2018). Lessons learned from implementing an incident command system during a local multiagency response to a legionnaires’ disease cluster in Sydney, NSW. Disaster Med. Public Health Prep..

[14] Kim J., Ashihara K. (2020). National disaster management system: COVID-19 case in Korea. Int. J. Environ. Res. Public Health.

[15] Dynes R.R. (1994). Community emergency planning: False assumptions and inappropriate analogies. Int. J. Mass Emerg. Disasters.

[16] Wenger D., Quarantelli E.L., Dynes R.R. (1990). Is the incident command system a plan for all seasons and emergency situations. Hazard Mon..

[17] Christensen T., Andreas Danielsen O., Laegreid P., Rykkja H.L. (2016). Comparing coordination structures for crisis management in six countries. Public Adm..

[18] Ang Y.Y. (2020). When COVID-19 meets centralized, personalized power. Nat. Hum. Behav..

[19] Hermansson H.M. (2016). Disaster management collaboration in Turkey: Assessing progress and challenges of hybrid network governance. Public Adm..

[20] Lutz L.D., Lindell M.K. (2008). Incident command system as a response model within emergency operation centers during Hurricane Rita. J. Contingencies Crisis Manag..

[21] Comfort L.K. (1994). Self-organization in complex systems. J. Public Adm. Res. Theory.

[22] Jensen J., Thompson S. (2016). The incident command system: A literature review. Disasters.

[23] Ministry of Health and Welfare (2016). The 2015 MERS Outbreak in the Republic of Korea: Learning from MERS.

[24] (2021). World Health Organization MERS Situation Update. https://applications.emro.who.int/docs/WHOEMCSR417E-eng.pdf?ua=1.

[25] Borgatti S.P., Everett M.G., Freeman L.C. (2002). Ucinet for Windows: Software for Social Network Analysis.

[26] Wasserman S., Faust K. (1994). Social Network Analysis: Methods and Applications.

[27] Krackhardt D., Carley K.M., Prietula M.J. (1994). Graph theoretical dimensions of informal organizations. Computational Organization Theory.

[28] Krackhardt D., Stern R.N. (1988). Informal networks and organizational crises: An experimental simulation. Soc. Psychol. Q..

[29] Bae Y., Joo Y.-M., Won S.-Y. (2016). Decentralization and collaborative disaster governance: Evidence from South Korea. Habitat Int..

[30] Kapucu N., Garayev V. (2016). Structure and network performance: Horizontal and vertical networks in emergency management. Adm. Soc..

[31] Choi S., Park J., Lee J. (2015). Refusal of Patient Transportation from Other Provinces: “Not in My Backyard”. https://m.khan.co.kr/view.html?art_id=201506052146395.

[32] Moon M.J. (2020). Fighting COVID-19 with agility, transparency, and participation: Wicked policy problems and new governance challenges. Public Adm. Rev..

[33] Ibarra H. (1999). Provisional selves: Experimenting with image and identity in professional adaptation. Adm. Sci. Q..

[34] Nowell B., Steelman T. (2015). Communication under fire: The role of embeddedness in the emergence and efficacy of disaster response communication networks. J. Public Adm. Res. Theory.

